# The COSMIN checklist for evaluating the methodological quality of studies on measurement properties: A clarification of its content

**DOI:** 10.1186/1471-2288-10-22

**Published:** 2010-03-18

**Authors:** Lidwine B Mokkink, Caroline B Terwee, Dirk L Knol, Paul W Stratford, Jordi Alonso, Donald L Patrick, Lex M Bouter, Henrica CW de Vet

**Affiliations:** 1 Department of Epidemiology and Biostatistics and the EMGO Institute for Health and Care Research, VU University Medical Center, Amsterdam, the Netherlands; 2 School of Rehabilitation Science and Department of Clinical Epidemiology and Biostatistics, McMaster University, Hamilton, Canada; 3 Health Services Research Unit, Institute Municipal d'Investigació Mèdica (IMIM-Hospital del Mar), Barcelona, Spain; 4 Centro de Investigación Biomédica en Red de Epidemiología y Salud Pública (CIBERESP), Spain; 5 Department of Health Services, University of Washington, Seattle, USA; 6 Executive Board of VU University Amsterdam, Amsterdam, the Netherlands

## Abstract

**Background:**

The COSMIN checklist (COnsensus-based Standards for the selection of health status Measurement INstruments) was developed in an international Delphi study to evaluate the methodological quality of studies on measurement properties of health-related patient reported outcomes (HR-PROs). In this paper, we explain our choices for the design requirements and preferred statistical methods for which no evidence is available in the literature or on which the Delphi panel members had substantial discussion.

**Methods:**

The issues described in this paper are a reflection of the Delphi process in which 43 panel members participated.

**Results:**

The topics discussed are internal consistency (relevance for reflective and formative models, and distinction with unidimensionality), content validity (judging relevance and comprehensiveness), hypotheses testing as an aspect of construct validity (specificity of hypotheses), criterion validity (relevance for PROs), and responsiveness (concept and relation to validity, and (in) appropriate measures).

**Conclusions:**

We expect that this paper will contribute to a better understanding of the rationale behind the items, thereby enhancing the acceptance and use of the COSMIN checklist.

## Background

For the measurement of health-related patient-reported outcomes (HR-PROs) it is important to evaluate the methodological quality of studies in which the measurement properties of these instruments are assessed. When studies on measurement properties have good methodological quality, their conclusions are more trustworthy. A checklist containing standards for design requirements and preferred statistical methods is a useful tool for this purpose. However, there is not much empirical evidence for the content of such a tool. A Delphi study is a useful study design in fields lacking empirical evidence. It is particularly valued for its ability to structure and organize group communication [1].

In an international Delphi study we developed the COSMIN (COnsensus-based Standards for the selection of health status Measurement INstruments) checklist for evaluating the methodological quality of studies on measurement properties [2,3]. The checklist contains twelve boxes. Ten boxes can be used to assess whether a study meets the standards for good methodological quality. Nine of these boxes contain standards for the included measurement properties (internal consistency, reliability, measurement error, content validity (including face validity), structural validity, hypotheses testing, and cross-cultural validity (these three are aspects of construct validity), criterion validity, and responsiveness), and one box contains standards for studies on interpretability. In addition, two boxes are included in the checklist that contain general requirements for articles in which IRT methods are applied (IRT box), and general requirements for the generalisability of the results (Generalisability box), respectively. More information on how to use the COSMIN checklist can be found elsewhere [4].

The checklist can be used, for example, in a systematic review of measurement properties, in which the quality of studies on measurement properties of instruments with a similar purpose are assessed, and results of those studies are compared with a view to select the best instrument. If the results of high-quality studies differ from the results of low-quality studies, this can be an indication of bias. Consequently, instrument selection should be based on the high-quality studies. The COSMIN checklist can also be used as guidance for designing or reporting a study on measurement properties. Furthermore, students can use the checklist when learning about measurement properties, and reviewers or editors of journals can use it to appraise the methodological quality of studies on measurement properties. Note that the COSMIN checklist is not a checklist for the evaluation of the quality of a HR-PRO, but for the methodological quality of studies on their measurement properties.

As a foundation for the content of the checklist, we developed a taxonomy of all included measurement properties, and reached international consensus on terminology and definitions of measurement properties [5]. The focus of the checklist is on studies on measurement properties of HR-PROs used in an evaluative application, i.e. longitudinal assessment of treatment effects or changes in health over time.

In this paper, we provide a clarification for some parts of the COSMIN checklist. We explain our choices for the included design requirements and preferred statistical methods for which no evidence is available in the literature or which generated substantial discussion among the members of the Delphi panel. The topics that are subsequently discussed in detail are internal consistency, content validity, hypotheses testing as an aspect of construct validity, criterion validity, and responsiveness.

### Internal Consistency

**Figure 1:**
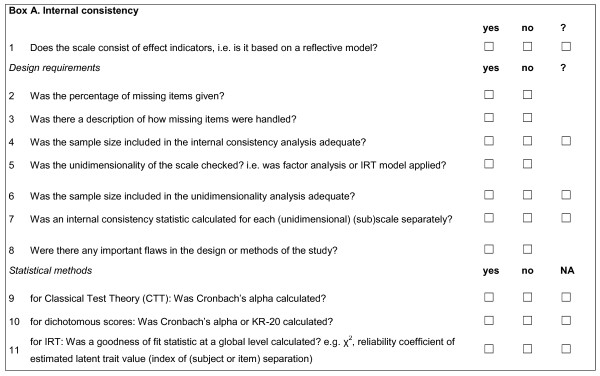
**Box A**. Internal consistency.

Internal consistency was defined as the interrelatedness among the items [5]. In Figure 1 its standards are given. The discussion was about the relevance of internal consistency for reflective models and formative models, and on the distinction between internal consistency and unidimensionality.

The Delphi panel reached consensus that the internal consistency statistic only gets an interpretable meaning, when (1) the interrelatedness among the items is determined of a set of items that together form a reflective model, and (2) all items tap the same construct, i.e., they form a unidimensional (sub)scale [6,7].

A reflective model is a model in which all items are a manifestation of the same underlying construct [8,9]. These items are called effect indicators and are expected to be highly correlated and interchangeable [9]. Its counterpart is a formative model, in which the items together form a construct [8]. These items do not need to be correlated. Therefore, internal consistency is not relevant for items that form a formative model. For example, stress could be measured by asking about the occurrence of different situations and events that might lead to stress, such as job loss, death in a family, divorce etc. These events obviously do not need to be correlated, thus internal consistency is not relevant for such an instrument. Often, authors do not explicitly describe whether their HR-PRO is based on a reflective or formative model. To decide afterwards which model was used, one can do a simple "thought test". With this test one should consider whether *all *item scores are expected to change when the construct changes. If yes, a reflective model is at issue. If not, the HR-PRO instrument is probably based on a formative model [8].

For an internal consistency statistic to get an interpretable meaning the scale needs to be unidimensional. Unidimensionality of a scale can be investigated with e.g. a factor analysis, but not with an assessment of internal consistency [8]. Rather, unidimensionality of a scale is a prerequisite for a clear interpretation of the internal consistency statistics [6,7].

### Content validity

**Figure 2:**
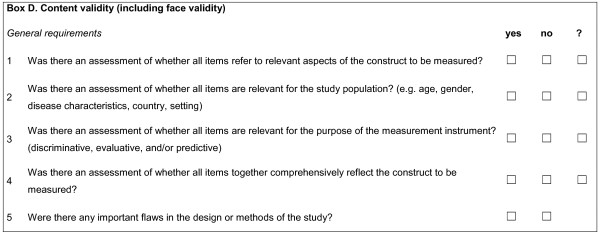
**Box D**. Content validity (including face validity).

Content validity was defined as the degree to which the content of a HR-PRO instrument is an adequate reflection of the construct to be measured [5] (see Figure 2 for its standards). The discussion was about how to evaluate content validity.

The Delphi panel agreed that content validity should be assessed by making a judgment about the *relevance *and the *comprehensiveness *of the items. The *relevance *of the items should be assessed by judging whether the items are relevant for the construct to be measured (D1), for the study population (D2), and for the purpose of the HR-PRO (D3). When a new HR-PRO is developed, the focus and detail of the content of the instrument should match the target population (D2). When the instrument is subsequently used in another population than the original target population for which it was developed, it should be assessed whether all items are relevant for this new study population (D2). For example, a questionnaire measuring shoulder disability (i.e., the Shoulder Disability Questionnaire [10]) may include the item "my shoulder hurts when I bring my hand towards the back of my head". When one decides to use this questionnaire in a population of patients with wrist problems to measure wrist disability, one could not simply change the word "shoulder" into "wrist" because this item might not be relevant for patients with wrist problems. Moreover, an item like "Do you have difficulty with the grasping and use of small objects such as keys or pens?" [11] will probably not be included in a questionnaire for shoulder disability, while it is clearly relevant to ask patients with wrist problems.

*Experts*, should judge the relevance of the items for the construct (D1), for the patient population (D2), and for the purpose (D3). Because the focus is on PROs *patients *should be considered as experts when judging the relevance of the items for the patient population (D2). In addition, many missing observations on an item can be an indication that the item is not relevant for the population, or it is ambiguously formulated.

To assess the *comprehensiveness *of the items (D4) three aspects should be taken into account: the content coverage of the items, the description of the domains, and the theoretical foundation. The first two aspects refer to the question if *all *relevant aspects of the construct are covered by the items and the domains. The theoretical foundation refers to the availability of a clear description of the construct, and the theory on which it is based. A part of this theoretical foundation could be a description of how different constructs within a concept are interrelated, like for instance as described in the model of health status of Wilson and Cleary [12] or the International Classification of Functioning, Disability and Health (ICF) model [13]. An indication that the comprehensiveness of the items was assessed could be that patients or experts were asked whether they missed items. Large floor and ceiling effects can be an indication that a scale is not comprehensive.

### Construct validity

Construct validity is the degree to which the scores of an HR-PRO instrument are consistent with hypotheses *(for instance with regard to internal relationships, relationships to scores of other instruments, or differences between relevant groups) *based on the assumption that the HR-PRO instrument validly measures the construct to be measured [5]. It contains three aspects, i.e. structural validity, which concerns the internal relationships, hypotheses testing, and cross-cultural validity, which both concern the relationships to scores of other instruments, or differences between relevant groups.

#### Hypotheses testing

**Figure 3:**
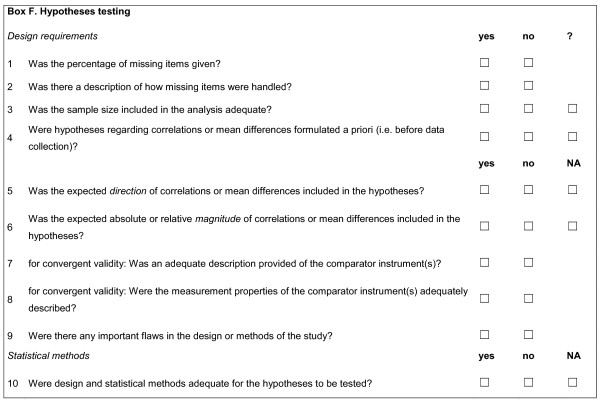
**Box F**. Hypotheses testing.

The standards for hypotheses testing are given in Figure 3. The discussion was about how specific the hypotheses that are being formulated should be.

Hypotheses testing is an ongoing, iterative process [14]. Specific hypotheses should include an indication of the expected direction and magnitude of correlations or differences. Hypotheses testing is about whether the direction and magnitude of a correlation or difference is similar to what could be expected based on the construct(s) that are being measured. The more hypotheses are being tested on whether the data correspond to a priori formulated hypotheses, the more evidence is gathered for construct validity.

The Delphi panel agreed that specific hypotheses to be tested should be formulated a priori (F4) about expected mean differences between known groups or expected correlations between the scores on the instrument and other variables, such as scores on other instruments, or demographic or clinical variables. The expected direction (positive or negative) (F5) and magnitude (absolute or relative) (F6) of the correlations or differences should be included in the hypotheses (e.g. [14-17]).

For example, an investigator may theorize that two HR-PROs intended to assess the same construct should correlate. Therefore, the investigator would test whether the observed correlation equals the expected correlation (e.g. > 0.70). The hypotheses may also concern the relative magnitude of correlations, for example "it is expected that the score on measure A correlates higher (e.g. 0.10 higher) with the score on measure B than with the score on measure C".

A hypothesis can also concern differences in scores between groups. When assessing differences between groups, it is less relevant whether these differences are statistically significant (which depends on the sample size) than whether these differences have the expected magnitude. For example, an investigator may theorize based on previous evidence that persons off work with low back pain have more pain related disability than persons working with low back pain. Accordingly, an instrument measuring pain related disability would be valid in this context if it is capable to distinguish these two groups. However, it is preferable to specify a minimally important between-group difference. The Delphi panel recommended that p-values should be avoided in the hypotheses, because it is not relevant to examine whether correlations or differences statistically differ from zero [18]. The size of the difference is more important than significant differences between the groups, since this is dependent on the number of subjects in each group. Formal hypotheses testing is preferable based using the expected magnitude of correlations and differences, rather than p-values.

When hypotheses are formulated about expected relations with other instruments, these comparator instruments should be appropriately described (F7). For example, if the comparator instrument intends to measure physical activity (PA), it should be described which construct exactly it aims to measure. Some PA instruments aim to measure total energy expenditure, others are focussed on duration of physical activities, on the frequency of activities, or on type of activities [19]. Ideally, the measurement properties of the comparator instruments should have been assessed in the same language version and the same patient population as is used in the study.

### Criterion validity

Criterion validity was defined as the degree to which the scores of a HR-PRO instrument are an adequate reflection of a "gold standard" [5]. The criterion used should be considered as a reasonable "gold standard" (H4). The Delphi panel reached consensus that no gold standards exist for HR-PRO instruments, and discussed whether criterion validity should be mentioned at all in the COSMIN checklist. The panel decided that the only exception of a gold standard is when a shortened instrument is compared to the original long version. In that case, the original long version can be considered the gold standard. Often, authors consider their comparator instrument wrongly as a gold standard, for example when they compare the scores of a new instrument to a widely used instrument like the SF-36. When the new instrument is compared to the SF-36, we consider it as construct validation, and expected hypotheses about the magnitude and direction of the correlation between (subscales of) the instruments should be formulated and tested.

### Responsiveness

The discussion on responsiveness was about the concept of responsiveness, (in)appropriate methods to evaluate responsiveness, and its relationship with validity. In the COSMIN study responsiveness was defined as the ability of a HR-PRO instrument to detect change over time in the construct to be measured [5]. Although the Delphi panel wanted to discuss responsiveness as a separate measurement property, the panel agreed that the only difference between cross-sectional (construct and criterion) validity and responsiveness is that validity refers to the validity of a single score, and responsiveness refers to the validity of a change score [5]. Therefore, the panel decided that the standards for responsiveness should be analogue to the standards for construct and criterion validity. Similarly as with criterion validity, it was agreed that no gold standards exist for change scores on HR-PROs, with the exception of change on the original longer version of a HR-PRO that can be considered a gold standard, when it is compared to change on its shorter version.

Appropriate measures to evaluate responsiveness are the same as those for hypotheses testing and criterion validity, with the only difference that hypotheses should focus on the change score of an instrument. For example, De Boer et al. assessed responsiveness of the Low Vision Quality of Life questionnaire (LVQOL) and the Vision-Related Quality of Life Core Measure (VCM1) by testing pre-specified hypotheses about the relations of changes in the questionnaires with changes in other measures in patient with irreversible vision loss [20]. They hypothesized, for example, that 'the correlation of change on the LVQOL/VCM1 with change on the Visual Functioning questionnaire (VF-14) is higher than the correlation with the global rating scale, change in visual acuity and change on the Euroqol thermometer'. After calculating correlations between the change scores on the different measurement instruments they concluded whether the correlations were as expected.

There are a number of parameters proposed in the literature to assess responsiveness that the Delphi panel considers inappropriate. The panel reached consensus that the use of effect sizes (mean change score/SD baseline) [21], and related measures, such as standardised response mean (mean change score/SD change score) [22], Norman's responsiveness coefficient (σ^2 ^change/σ^2 ^change + σ^2 ^error) [23], and relative efficacy statistic ((t-statistic_1_/t-statistic_2_)^2^) [24] are inappropriate measures of responsiveness. The paired t-test was also considered to be inappropriate, because it is a measure of significant change instead of valid change, and it is dependent on the sample size of the study [18]. These measures are considered measures of the magnitude of change due to an intervention or other event, rather than measures of the quality of the measurement instrument [25,26]. Guyatt's responsiveness ratio (MIC/SD change score of stable patients) [27] was also considered to be inappropriate, because it takes the minimal important change into account. The Delphi panel agreed that minimal important change concerns the interpretation of the change score, but not the validity of the change score.

## Discussion

In this article, we explained our choices for the design requirements and preferred statistical methods for which no evidence is available in the literature or which generated major discussions among the members of the Delphi study during the development of the COSMIN checklist. However, within the four rounds of the Delphi study, two issues could not be discussed extensively, due to lack of time. These issues concerned factor analyses (mentioned in Box A internal consistency and Box E structural validity) and minimal important change (mentioned in Box J interpretability).

The Delphi panel decided that the evaluation of structural validity can be done either by explorative factor analysis or confirmative factor analysis. However, confirmatory factor analysis is preferred over explorative factor analysis, because confirmative factor analysis tests whether the data fit an a priori hypothesized factor structure [28], while explorative factor analysis can be used when no clear hypotheses exist about the underlying dimensions [28]. Such an explorative factor analysis is not a strong tool in hypothesis testing. In the COSMIN study we did not discuss specific requirements for factor analyses, such as the choice of the explorative factor analysis (principal component analysis or common factor analysis), the choice and justification of the rotation method (e.g. orthogonal or oblique rotation), or the decision about the number of relevant factors. Such specific requirements are described by e.g. Floyd & Widaman [28] and De Vet et al. [29].

In the Delphi panel it was discussed that in a study evaluating the interpretability of scores of an HR-PRO instrument the minimal important change (MIC) or minimal important difference (MID) should be determined. The MIC is the smallest change in score in the construct to be measured which patients perceive as important. The MID is the smallest differences in the construct to be measured between patients that is considered important [30]. Since we talk about *patient*-reported outcomes, the agreement among panel members was that the patients should be the one to decide on what is important. In the literature there is an ongoing discussion about which methods should be used to determine the MIC or MID of a HR-PRO instrument [31]. Consequently, the opinions of the panel members differed widely, and within the COSMIN study no consensus on standards for assessing MIC could be reached.

The results of a Delphi study are dependent on the composition of the panel. The panel members do not need to be randomly selected to represent a target population. Rather experts are chosen because of their knowledge of the topic of interest [32,33]. It has been noted that heterogeneous groups produce a higher proportion of high-quality, highly acceptable solutions than homogeneous groups [1]. Furthermore, anonymity of each of the panel members is often recommended, because it provides an equal chance for each panel member to present and react to ideas unbiased by the identities of other participants [34]. Both issues were ensured in this Delphi study. We included experts in the field of psychology, epidemiology, statistics and clinical medicine. The panel members did not know who the other panel members were. All questionnaires were analysed and reported back anonymously. Only one of the researchers (LM) had access to this information.

The COSMIN Delphi study focussed on assessing the methodological quality of studies on measurement properties of existing HR-PROs. However, we think that the discussions described above and the COSMIN checklist itself are also relevant and applicable for researchers who are developing HR-PROs. The COSMIN checklist can be a useful tool for designing a study on measurement properties.

## Conclusions

In conclusion, as there is not much empirical evidence for standards for the assessment of measurement properties, we consider the Delphi technique the most appropriate method to develop a checklist on the methodological quality of studies on measurement properties. Within this Delphi study we have had many interesting discussions, and reached consensus on a number of important issues about the assessment of measurement properties. We expect that this paper will contribute to a better understanding of the rationale behind the items in the COSMIN checklist, thereby enhancing its acceptance and use.

## Competing interests

The authors declare that they have no competing interests.

## Authors' contributions

CT and HdV secured funding for the study. CT, HdV, LB, DK, DP, JA, and PS conceived the idea for the study. LM and CT prepared all questionnaires for the four Delphi rounds, supervised by HdV, DP, JA, PS, DK and LB. LM, CT, and HdV interpreted the data. LM coordinated the study and managed the data. CT, DP, JA, PS, DK, LB and HdV supervised the study. LM wrote the manuscript with input from all the authors. All authors read and approved the final version of the report.

## Pre-publication history

The pre-publication history for this paper can be accessed here:

http://www.biomedcentral.com/1471-2288/10/22/prepub
